# Canada’s Decentralized “Human-Driven” Approach During the Early COVID-19 Pandemic

**DOI:** 10.2196/20343

**Published:** 2020-12-23

**Authors:** Gregory Hansen, Amelie Cyr

**Affiliations:** 1 Jim Pattison Children's Hospital Saskatoon, SK Canada; 2 Department of Pediatrics College of Medicine University of Saskatchewan Saskatoon, SK Canada

**Keywords:** COVID-19, coronavirus infection, public health

## Abstract

A country’s early response to a pandemic is critical for controlling the disease outbreak. During the COVID-19 pandemic, a number of southeast Asian countries adopted centralized, coordinated, rapid, and comprehensive approaches that involved smart technology (the “techno-driven” approach). In comparison, Canada’s approach appeared to be decentralized, uncoordinated, and slow, and it focused on educating citizens and enhancing social and human capital (the “human-driven” approach). We propose that in future pandemics, early and coordinated “techno-driven” approaches should receive more careful consideration to curtail outbreaks; however, these approaches must be balanced with protecting individuals’ freedoms.

## Introduction

On December 31, 2019, the World Health Organization (WHO) was alerted to a pneumonia of unknown cause in Wuhan, China [1]. In January 2020, a novel coronavirus called SARS-CoV-2 was identified, its RNA was sequenced, and a public health emergency of international concern was declared. In February, the WHO announced COVID-19 as the name of disease caused by the new coronavirus, and one month later, it characterized the outbreak of COVID-19 as a pandemic [1].

For years, the WHO has provided global leadership for pandemic preparedness and response. This organization has recognized that early and effective planning can attenuate social and economic disruption, threats to essential services, and difficulties with production levels, distribution, and shortages of supplies [2]. Consequently, the WHO has created a comprehensive framework that guides national actions with planning and coordination, pandemic disease surveillance, monitoring impact (ie, medical supplies), reducing spread of disease, and communications [3]. Inherent to the framework is the capacity to determine the pathogen’s effect as early as possible so that the proportionate response can be executed [4,5]. Determining disease severity and transmissibility through early identified cases, known as “first few hundred” studies, is key [5].

Evaluating early national responses to their first few hundred COVID-19 cases may be useful to optimize future global pandemic actions. Canada’s 1st case was reported on January 25, 2020 [6], and 45 days later, its 99th case was announced. Canada’s response during this critical time largely employed a “human-driven” approach, which relied on educating citizens and enhancing social and human capital [7].

In this viewpoint, we are critical of Canada’s “human-driven” approach by contrasting it with key recommendations of the WHO’s pandemic document [3] and “techno-driven” approaches that were effective in other countries. A “techno-driven approach” relies on top-down initiatives that mandate widespread use of smart technology [7], and it includes measures such as contact tracing apps and data collection surveillance.

## Planning and Coordination

Ironically, one of Canada’s most important global contributions to global health was in the midst of a long-overdue upgrade during the initial stages of the COVID-19 pandemic. The Global Public Health Intelligence Network (GPHIN) is an all-hazards software surveillance system developed by the federal government of Canada to provide situational awareness by collecting and analyzing new articles, incident reports, and media releases from multiple sources and languages [8]. The GPHIN is credited with helping identify the severe acute respiratory syndrome (SARS) and H1N1 influenza outbreaks, and the WHO is one of its many subscribers; however, its effectiveness may have been attenuated by an outdated algorithm and limited data sources [9]. Instead, a private Canadian software company called BlueDot was not only the first to warn of the new illness [10] but also predicted the next 11 cities that would be affected. Because inadequate funding and an aging network have both been cited as challenges for the GPHIN, collaborating with nongovernmental big data research centers may be an alternative for the future.

On January 15, 2020, when only a few cases of COVID-19 had been reported globally, the Public Health Agency of Canada (PHAC) triggered the federal, provincial, and territorial Public Health Response Plan for Biological Events and activated the Health Portfolio Operations Centre [11]. The former was intended to facilitate efficient, evidence-based, timely, consistent, and coordinated approaches across jurisdictions, while the latter acted as the point of contact for operational communications and emergency management governance support [12]. As COVID-19 spread globally, its identity began to take shape: asymptomatic carrier transmission (February 21) [13], basic reproduction numbers ranging from 1.4 to 3.11 (January 23) [14,15], a case fatality ratio of 3.5%, and a mean incubation period of approximately 5 days (February 17) [16].

Despite the GPHIN setback, it is evident that early during its first 100 cases, Canada’s public health system was assembled and aware. A gradual federal stepwise response ensued (Table 1), and the Canadian Public Health Response Plan for Biological Events was heightened to Level Three—“Escalated.”

However, despite the continuation of significant interprovincial air, train, road, and marine transportation and travel, a coordinated provincial/territorial response did not follow, and public health actions were initiated on different dates or not at all (Table 2).

**Table 1:** Early response to the COVID-19 pandemic by the federal government of Canada.

Date (2020)	Federal government interventions	China cases (new)	China deaths (new)	Global cases (new)	Global deaths (new)	Countries/territories/areas	Canada cases (new)	Canada deaths (new)	Canada tests (per million)
Jan 15	PHAC^a^ activated Emergency Operations Centre	41	2 (1)	1	0	2	0	0	0
Jan 22	Screening for travelers returning from China	571 (131)	17 (11)	9	0	8	0	0	0
Feb 9	Screening for travelers returning from affected areas (10 airports, 6 provinces)	40,171 (2973)	908 (97)	382 (28)	2 (0)	26	7 (0)	0	346 (0.00000913)
Feb 27	Escalation to Level Three Response Level (Public Health Response Plan for biologic events)	78,824 (327)	2788 (44)	4228 (905)	70 (14)	53	14 (2)	0	1663 (0.000044)
Mar 13	Advised avoiding all nonessential travel outside of Canada	80,824 (11)	3189 (13)	64,592 (10,896)	2239 (434)	136	198 (56)	1 (0)	21,251 (0.00056)
Mar 16	Advised travelers entering Canada to self-isolate for 14 days	80,881 (21)	3226 (13)	101,533 (12,876)	3936 (629)	160	441 (100)	4 (3)	40,935 (0.0011)
Mar 18	Banned foreign nationals from all countries (except the United States); closed US-Canada border; redirected all international passenger flight arrivals to 4 airports; announced financial help	80,928 (34)	3245 (8)	137,816 (20 551)	5706 (964)	177	727 (129)	9 (1)	60,845 (0.0016)
Mar 25	Quarantine Act mandated all returning travelers to isolate themselves for 14 days	81,285 (67)	3287 (6)	389,750 (48,394)	17,995 (2382)	198	3409 (617)	36 (3)	138,700 (0.0037)

^a^PHAC: Public Health Agency of Canada

**Table 2:** Early responses to the COVID-19 outbreak by provincial and territorial governments.

Action	Province or territory^a^												
	BC [17]	AB [18]	SK [19]	MB [20]	ON [21]	QC [22,23]	NB [24]	NS [25]	PE [26]	NL [27]	YT [28]	NT [29]	NU [30]
Self-isolation advised if returning from China	Feb 4	Feb 6	Feb 13	Feb 7	Feb 6	Feb 8	Feb 7	Feb 6	Feb 28	Feb 6	Feb 7	Mar 10	N/A^b^
Self-isolation advised if returning from a cruise	Feb 19	Mar 5	N/A	N/A	N/A	Mar 10	N/A	N/A	N/A	N/A	N/A	Mar 10	N/A
Self-monitoring of symptoms advised if traveled anywhere	Feb 24	Mar 6	Mar 11	Feb 27	Mar 10	Mar 11	Mar 1	Mar 9	Mar 9	Mar 6	Mar 13	Mar 14	Mar 4
Self-isolation advised if returning from Iran and China	Mar 3	Mar 2	Mar 5	Mar 5	Mar 3	Mar 3	Mar 9	Mar 13	N/A	N/A	Mar 13	Mar 10	N/A
Self-isolation advised if returning from Italy	N/A	Mar 12	Mar 9	Mar 12	Mar 13	Mar 10	Mar 11 (health care workers only)	Mar 13	N/A	N/A	Mar 13	Mar 14	N/A
Self-isolation advised if returning from outside of Canada	Mar 12	Mar 12	Mar 14	Mar 15	Mar 14	Mar 12	Mar 19	Mar 15	Mar 13	Mar 14	Mar 16	Mar 15	Mar 15 (announced Mar 18)
Screening event participants (symptoms, international travelers)	Mar 3	Mar 10	Mar 16	N/A	N/A	N/A	N/A	N/A	N/A	N/A	N/A	N/A	N/A
Social distancing recommended	Mar 7	Mar 13	Mar 11	Mar 12	Mar 15	Mar 12	Mar 12	Mar 13	Mar 13	Mar 13	Mar 16	Mar 17	Mar 13
Testing announced for travelers to at-risk areas and for local transmission	Jan 21	Feb 26	Feb 13	Jan 28	Jan 24	Jan 22	Feb 10	Feb 6	Feb 28	Feb 6	Jan 14	Feb 5	N/A
Testing expanded to any international traveler with symptoms and contacts	Feb 25	Mar 6	Mar 11	Feb 27	Feb 28	Mar 8	Mar 1	Mar 9	Mar 9	Mar 6	Mar 16	Mar 14	N/A
All respiratory specimens tested for COVID-19	N/A	Mar 12	NA	Mar 12	Mar 5	N/A	N/A	N/A	N/A	N/A	N/A	N/A	N/A
First testing center(s) opened	Mar 13	Mar 15	Mar 13	Mar 12	Mar 13	Mar 9	Mar 16	Mar 11	Mar 11	Mar 21	Mar 26	Mar 23 (drive-through)	N/A

^a^Abbreviations: BC: British Columbia; AB: Alberta; SK: Saskatchewan; MB: Manitoba; ON: Ontario; QC: Quebec: NB: New Brunswick; NS: Nova Scotia; PE: Prince Edward Island; NL: Newfoundland and Labrador; YT: Yukon Territory; NT: Northwest Territories; NU: Nunavut.

^b^N/A: not applicable.

## Pandemic Disease Surveillance

The WHO recommends a comprehensive assessment of the earliest cases, with documentation of geographical spread, trends, and impact, to better inform public health response [3]. Canada prioritized the protection of individuals’ privacy, collected volunteered data, and relied on coordination between national and provincial or territorial departments [7]. On February 10, PHAC provided interim surveillance guidelines with COVID-19 case definitions to guide provincial/territorial legislation, regulations, and policies [31]. This included reporting cases nationally to PHAC within 24 hours of notification, provincial/territorial case interviews to identify close contacts, and monitoring health status. Identified close contacts were categorized based on risk exposure, with high risks requiring intermittent public health monitoring only.

During the first 100 cases, concluding on March 10, Canada performed approximately 11,023 tests, with significant variation between provinces and territories. The tests were initially processed at Canada’s national laboratory in Winnipeg, an inefficient process that led to delayed reporting. By February 25, only provincial laboratories in Ontario and British Columbia were ready to perform independent testing; however, routine COVID-19 testing for respiratory specimens was not implemented until later. The Canadian Broadcasting Corporation reported on testing variations and noted provincial/territorial differences in rates of testing, groups that were “targeted,” and frequent changes to policies [32]. Together, these actions led to large exclusions of asymptomatic and even symptomatic patients outside the “targeted” groups [32].

Buoyed by lessons learned from previous pandemics, South Korea launched an expansive and organized surveillance program that could objectively verify contact investigations. Recognizing that interviews would result in omitted or erroneous information, the importance of evaluating between-person risk and the elimination of infection exposure in contaminated places were prioritized. Interviews, medical facility records, credit card transactions, closed-circuit television and medical facility records were used to determine case locations, evaluate exposure risk, and classify contacts [33]. Social media apps were employed that informed users of potential contact with infected persons. During the first 100 cases in South Korea, 13,302 tests were conducted over a 30-day period [34]. Commercial test kits were rapidly developed, and drive-through screening centers were created early in the outbreak. This enabled increased testing capacity and prevention of cross-infection of testees by eliminating public waiting spaces [35].

South Korea’s techno-driven approach enabled a wider spectrum of societal surveillance and the capturing of cases well beyond the capacity of Canada’s human-driven approach, which focused on interviewing and testing hospitalized patients, health care workers, residents in long-term care facilities, or other “clusters.” Interestingly, although the approaches were somewhat polarizing regarding individual privacy versus public safety priorities, as the COVID-19 pandemic progressed in Canada, discussions around implementing techno-driven measures began to intensify.

## Reducing the Spread of COVID-19

### Individual- and Household-Level Measures

Individual- and household-level measures minimize interactions within and outside the home at the onset of symptoms [3]. During Canada’s first 100 cases, self-isolation was mainly recommended for positive cases, close contacts, and people returning from high-risk travel regions. Later, as the number of COVID-19 cases increased, an Emergency Order under Canada’s Quarantine Act legally mandated self-isolation after international travel, with violators facing significant financial penalties and/or imprisonment.

In contrast, Singapore’s experience of rapidly containing the SARS outbreak led to the quarantine of 425 close contacts at home or designated government facilities after only 36 cases from 3 clusters were identified [36]. “Close contacts” were identified as individuals who had spent a prolonged period of time within 2 meters of a confirmed case. “Other contacts,” or individuals who had some interactions with confirmed cases, were also followed. The health status and location of the contacts were routinely monitored via videoconferencing or telephone surveillance, clear directives were provided in the event that an individual became unwell, and quarantine violators were tagged with continuous tracking devices. These actions were deemed necessary to document early community transmission and facilitate containment efforts [36].

### Societal-Level Measures

Societal-level measures include social distancing with a focus on school suspensions, working pattern adjustments, reduction of crowding on public transportation, and cancellation, modification, or restriction of mass gatherings [3]. Only two provinces recommended social distancing during Canada’s first 100 cases; all the aforementioned social level measures were employed later.

In contrast, Hong Kong’s experience with SARS demonstrated the importance of early community measures to reduce population contacts [37]. Within days of the first reported case in Hong Kong, theme parks were closed, cross-border bus services were suspended, school reopening was postponed, and civil servants adopted flexible working arrangements, including work-from-home options [38]. These restrictions, together with other nonpharmaceutical interventions, were associated with reduced COVID-19 transmission [38].

### International Travel Measures

International travel measures have been very contentious during the pandemic, as the WHO recommended implementing exit screens and providing advice to travelers [3] but did not recommend border closures. The Canadian government did create a basic contact information form with the Canadian Border Security Agency; however, 31 of 2226 travelers from Hubei Province were referred to PHAC, and of these, only 3 were medically examined [39]. Travelers from affected areas were also screened (Table 1), although more than half of infected people would be undetectable and missed because of unknown exposure or lack of symptoms [40]. Self-isolation after travel was also advised by most provinces and territories in an incremental fashion; however, sizable discrepancies in initiation dates can be noted (Table 2). On February 27, an open letter from 23 Chinese-Canadian doctors urged for the implementation of stronger measures that included a 14-day quarantine for travelers returning from COVID-19 hotspots [41]. These measures, including escalation to closed international borders and restricted domestic travel, would be realized well after the first 100 cases were reported.

In contrast, Taiwan’s national command center, a response to the SARS outbreak, rapidly implemented border control measures [42]. Passengers completed a health questionnaire upon arrival, and by integrating big data from national health and immigration registries, officials were able to quickly classify infectious risk status based on flight origin and travel history [42]. Travelers with minimal risks received a mobile pass that facilitated faster immigration clearance, while others identified as high risk were screened for 26 viruses, placed in home quarantine, and monitored electronically for compliance [42]. Further actions, including flight and visitor visa restrictions, would ensue; however, these actions were secondary in importance to Taiwan’s early border response, which began on the day China disclosed its first case.

## Continuity and Provision of Health Care

### Infection Control and Personal Protective Equipment

According to the WHO, enhancing infection control practices and distributing personal protective equipment (PPE) are important actions during an early pandemic response [3]. During the first 100 cases, PHAC evaluated domestic supplies of PPE and began to conserve and coordinate supplies due to mounting market pressures [39]. Only later, after PHAC recognized that their stockpile was not adequate, were Canadian companies asked to adjust their production lines to begin manufacturing PPE.

In contrast, Taiwan actively bolstered the provision of medical supplies very early in the pandemic. Specifically for PPE, authorities halted their exports, acquired assembly lines to boost domestic production, mobilized military personnel to assist manufacturers, and established a central distribution system. Using a cloud computing system, Taiwan also developed a rationing system based on national health insurance data [43] to prevent hoarding of PPE. This was no small task. To ensure that the new application would not overwhelm the cloud, in which medical records were normally stored, 20 new servers were set up by engineers in one day.

### Mobilizing the Health Care System

Pandemic contingency plans that mobilize health systems, facilities, and workers [3] may be more complicated in countries such as Canada, where the federal government has limited authority in the management, delivery, and organization of services. The federal PHAC did trigger a provincial and territorial public health response plan. However, during the first 100 cases, a patchwork of decentralized provincial and territorial responses resulted rather than quick, decisive, and cohesive actions that could have mitigated risk [44].

In contrast, Singapore’s Ministry of Health coordinated their COVID-19 activities through centralized systems. At the onset of their response, they activated a crisis system that enabled daily text messaging to the country, provided two-way communication channels with hospital executives, epidemiologists, and operational workgroups, and facilitated cross-hospital information sharing [45]. Their hospital systems were prepared through routine mass infectious crisis simulations that involved staff at every level [45].

## Communications

Finally, communications with the public regarding disruptions, sources, and resources for medical needs as well as COVID-19 itself [3] were conducted largely by PHAC and provincial and territorial authorities. Early in the pandemic, press conferences often lacked clarity, problems of uniform messaging between provinces and the federal government were noted, and releases of aggregate case statistics were inconsistent in timing and details [46]. When questioned whether the federal government should take a more proactive role in messaging and publishing data, Deputy Prime Minister Chrystia Freeland responded that “Canada is not a highly centralized country” [46].

Singapore recognized social media’s contribution to information flow during the SARS epidemic through its volume of use and the ease of creating false narratives [47]. Since the first case was reported in Singapore, the government has provided daily updates on traditional platforms such as print media, broadcasts, town hall meetings, and, more importantly, websites and social media (eg, WhatsApp, Twitter, Facebook, Telegram) [47]. Due to its high penetration, the WhatsApp social media platform was upgraded with artificial intelligence translation, easy signup, fast updates, and end-to-end data encryption to manage its increased demand and the need to rapidly deploy information.

## Beyond the First 100 Cases

As the pandemic progressed beyond the first 100 cases, Canadian officials began to look towards more “techno-driven” approaches to control the spread of COVID-19. For example, one app named “COVID Alert” was developed to inform citizens of possible exposures [48]; however, although the app protected users’ privacy, its adoption was not widespread. Testing became more streamlined, with many jurisdictions providing drive-through facilities. International and provincial borders were closed, PPE production and supplies were bolstered, and societal measures were amplified. Canada’s later actions mimicked the very early interventions from southeast Asian countries but continued to be largely uncoordinated between provincial entities.

## Implications

Canada’s early decentralized “human-driven” approach resulted in inefficient testing, suboptimal disease containment, and an inadequately mobilized health care system. These observations have also been noted in other Western democracies that value protection of individuals’ privacy, consensus building, and information sharing [7]. A coordinated “techno-driven” approach offers several pragmatic advantages; however, concerns about freedom and individuals’ rights must be considered. For future pandemics, the challenge may be to intentionally develop “techno-driven” approaches to assist with coordinated national responses while protecting individuals’ privacies and freedoms.

## Conclusions

Canada’s response to the COVID-19 outbreak during its first 100 cases could be characterized as decentralized, uncoordinated, slow, and “human-driven.” In contrast, a number of southeast Asian nations and jurisdictions that had wrestled with significant and recent pandemics demonstrated early responses that were centralized, coordinated, rapid, comprehensive, and “techno-driven.” Although these regions shared borders with China, received high volumes of travelers from Wuhan, and became involved in the pandemic very early, their mortality rates were miniscule compared to that in Canada. To optimize future action, an early coordinated approach that is “techno-driven” could be considered by Canadian public health officials.

## Abbreviations

GPHIN: Global Public Health Intelligence Network
PHAC: Public Health Agency of Canada
PPE: Personal Protective Equipment
SARS: severe acute respiratory syndrome
WHO: World Health Organization
